# Identification of SARS-CoV-2 Variants and Their Clinical Significance in Hefei, China

**DOI:** 10.3389/fmed.2021.784632

**Published:** 2022-01-10

**Authors:** Xiao-wen Cheng, Jie Li, Lu Zhang, Wen-jun Hu, Lu Zong, Xiang Xu, Jin-ping Qiao, Mei-juan Zheng, Xi-wen Jiang, Zhi-kun Liang, Yi-fan Zhou, Ning Zhang, Hua-qing Zhu, Yuan-hong Xu

**Affiliations:** ^1^Department of Clinical Laboratory, The First Affiliated Hospital of Anhui Medical University, Hefei, China; ^2^Laboratory of Molecular Biology, Department of Biochemistry, Anhui Medical University, Hefei, China; ^3^College of Life Sciences, Anhui Medical University, Hefei, China; ^4^Da An Gene Co., Ltd., Sun Yat-sen University, Guangzhou, China; ^5^The Medicine and Biological Engineering Technology Research Center of the Ministry of Health, Guangzhou, China; ^6^Clinical Laboratory Center, Guangzhou Darui Biotechnology, Co., Ltd., Guangzhou, China; ^7^Division of Life Sciences and Medicine, Department of Pathology, The First Affiliated Hospital of USTC, University of Science and Technology of China, Hefei, China; ^8^Wellcome Centre for Anti-Infectives Research, School of Life Sciences, University of Dundee, Dundee, United Kingdom

**Keywords:** COVID-19, SARS-CoV-2, genomic surveillance, S-type strain, endemic

## Abstract

The ongoing coronavirus disease 2019 (COVID-19) pandemic represents one of the most exigent threats of our lifetime to global public health and economy. As part of the pandemic, from January 10 to March 10, 2020, severe acute respiratory syndrome coronavirus-2 (SARS-CoV-2) began to spread in Hefei (Anhui Province, China) with a total of 174 confirmed cases of COVID-19. During this period, we were able to gather critical information on the transmission and evolution of pathogens through genomic surveillance. Particularly, the objective of our study was to track putative variants of SARS-CoV-2 circulating in Hefei for the first time and contribute to the global effort toward elucidating the molecular epidemic profile of the virus. Patients who showed symptoms of COVID-19 were routinely tested for SARS-CoV-2 infections *via* RT-PCR at the First Affiliated Hospital of Anhui Medical University. Whole-genome sequencing was performed on 97 clinical samples collected from 29 confirmed COVID-19 patients. As a result, we identified a local novel single-nucleotide polymorphism site (10,380) harboring a G → T mutation (Gly → Val) in Hefei. Further phylogenetic network analysis with all the sequences of SARS-CoV-2 deposited in GenBank collected in East and Southeast Asia revealed a local subtype of S-type SARS-CoV-2 (*a1*) harboring a C → T synonymous mutation (Leu) at position 18,060 of *ORF1b*, likely representing a local SARS-CoV-2 mutation site that is obviously concentrated in Hefei and the Yangtze River Delta region. Moreover, clinical investigation on the inflammatory cytokine profile of the patients suggested that mutations at positions 18,060 (the shared variable site of subtype a1) and 28,253(harboring a C → T synonymous mutation, Phe) were associated with milder immune responses in the patients.

## Introduction

The ongoing coronavirus disease 2019 (COVID-19) pandemic caused by severe acute respiratory syndrome coronavirus-2 (SARS-CoV-2) represents the most pressing and challenging threat to the present global public health and economy (1–3). At the time of writing this manuscript (04 November 2021), 247,968,227 confirmed cases of COVID-19, including 5,020,204 deaths, had been reported worldwide by the World Health Organization (WHO) (4).

COVID-19 is an infectious disease officially named by the World Health Organization (WHO) on February 11, 2020. The typical symptoms of patients suffering from COVID-19 include fever, drycough, dyspnea, myalgia, fatigue, unaltered or decreased white blood cell count, and radiographic evidence of pneumonia. Severe cases of COVID-19 often present with difficulty in breathing within a week after getting infected by the virus and promptly develop acute respiratory distress syndrome, septic shock, etc., eventually leading to multiple organ failure. Whereas, mildly infected patients manifest low-grade fever or mild fatigue but no pneumonia. At present, the primary treatment regimen followed by clinicians include antiviral therapy, antibacterial drug therapy and multi-organ support. Extracorporeal membrane oxygenation is one of the key therapeutic strategies to treat severe cases (5–8). So far, there is no specific clinically validated antiviral drug available for the treatment of COVID-19 (5). The current knowledge of the disease indicates that COVID-19 patients generally experience lymphopenia and inflammatory cytokine storms in the severe stage of the disease, and further affect different molecular and cellular pathways leading to multiple organ damage (9–13). Therefore, determining the state of immune cells and the underlying molecular mechanism of cytokine production may be the key steps in designing effective treatment course. The treatment methods currently in clinical trials include using miRNA mimics to inhibit the production of cytokines and other proteins which bring about the “cytokine storm” (14, 15), immune modulation therapy based on mesenchymal stem cells (16). The combination therapy of MAS receptor agonists and angiotensin type II receptor agonists may synergistically prevent disease progression (17). Statistical studies have shown that COVID-19 is highly contagious and indiscriminate regarding age, sex, nationality, and ethnicity (https://coronavirus.jhu.edu/map.html). The rapid mutations acquired by the virus and diversified transmission routes have significantly contributed to the COVID-19 pandemic (5). Vaccine development is a key strategy to prevent widespread viral infections and reduce morbidity and mortality. However, the high mutation rate of this single-stranded RNA virus presents a serious challenge to develop effective vaccines against SARS-CoV-2. Its high mutation rate means that it can quickly adapt its mode of transmission, virulence, and immune evasion (18, 19). Current epidemiological evidence shows that since the end of 2020, the continuous evolution of SARS-CoV-2 has resulted in the emergence of novel mutations (20–22). Interestingly, the surge in COVID-19 cases coincides with the emergence of these specific virus variants (23–25). WHO has traced 11 new variants of SARS-CoV-2 to date, out of which Alpha (B•1•1•7), Beta (B•1•351), Gamma (P•1), Delta (B•1•617• 2) variants are enlisted as “Variants of Concern” (VOC), and Eta (B•1•525), lota (B•1•526), Kappa (B•1•617•1) and Lambda (C•37) as “Variants of Interest (VOI)” (26, 27). At present, the Delta variant has become the most dominant strain in the world. Delta variants contain 10 mutations, which mainly in the spike protein (27, 28). Studies have shown that the increased replication adaptability of the Delta variant of SARS-CoV-2 and the decreased sensitivity to neutralizing antibodies have led to the recent rapid and large-scale spread of the virus (29). Monitoring SARS-CoV-2 mutations and their respective frequencies is essential as drug and vaccine trials continue as these data may help decide about the administration of multi-drug combinations and redesign the therapeutic strategy. Therefore, there is an urgent need for collaboration to promote data sharing and expansion of international genome monitoring resources (30). High-throughput sequencing data empower researchers to establish the molecular epidemiological landscape of the infection and construct molecular phylogenetic trees (31, 32). The history of viral transmission at both local and global levels can be traced by comparing the viral genomes and constructing molecular phylogenetic trees. This greatly facilitates the understanding of viral transmission, emergence of variants and their mutation rates, which are critical for developing effective therapies and vaccines (33, 34).

**Figure 1:**
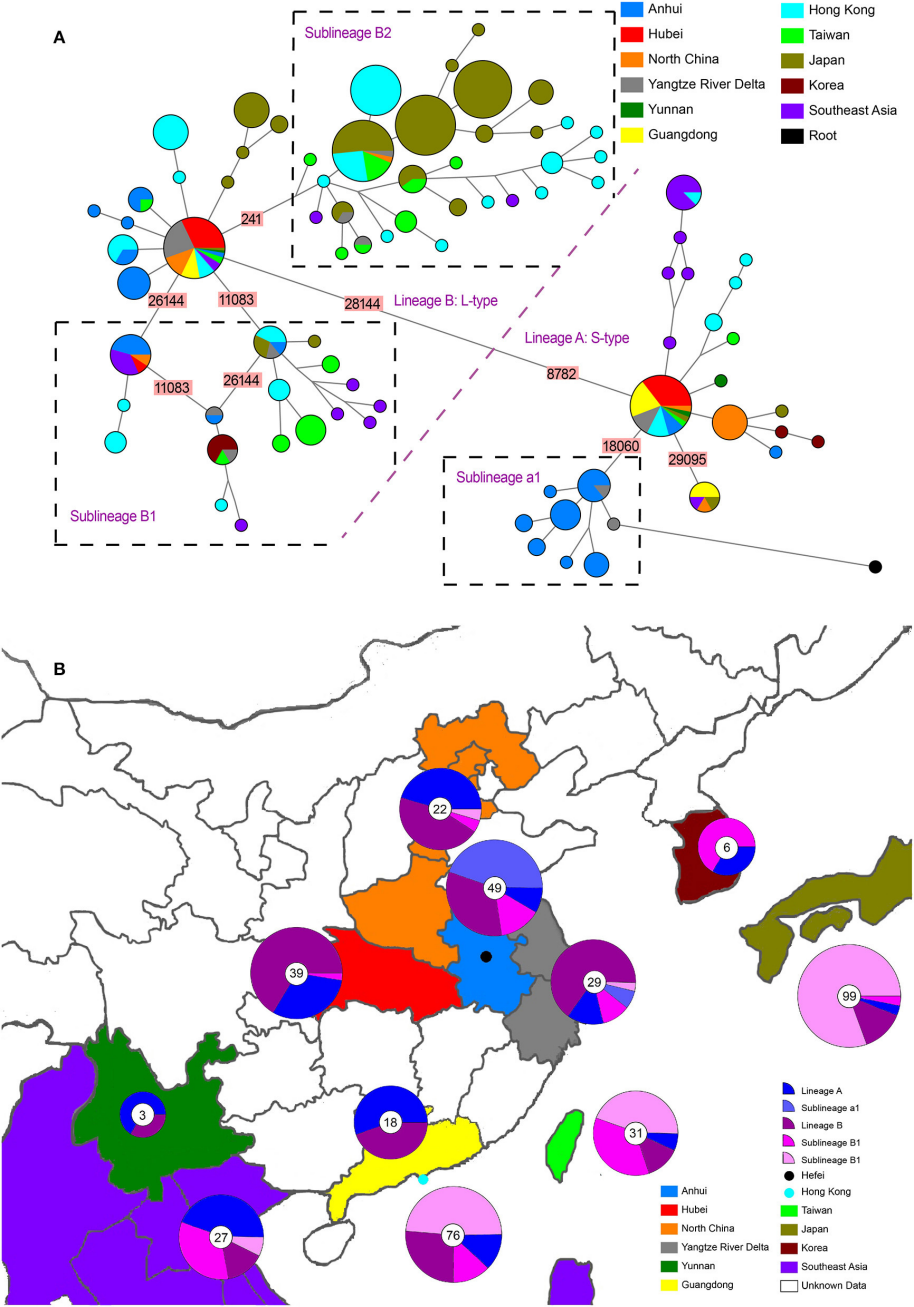
Phylogenetic network and geographical distribution of 48 SARS-CoV-2 genomes in study samples and 352 downloaded genome sequences. **(A)** Phylogenetic network. Circle areas are proportional to the number of sequences, and different colors represent different region of origin for each isolate. The edge linked two circle represent mutations between two sequences, several key site positions were highlighted by the number on the edge. Lineage A and B were separated by two mutations at T28144C and C8782T. The root was a bat coronavirus sequence (MN996532), which were the most closely related sequence to SARS-CoV-2 found in the wild. The sequences of Sublineage B1 had the same mutation on 26144 or 11083. The sequences of Sublineage B2 had the same mutation on 241. The sequences of Sublineage a1 had the same mutation on 18060. **(B)** Geographical distribution. Different colors in the map represent different geographical areas. The pie chart beside each area represent the frequency of different Lineage (or Sublineage). The number in the center of each pie chart represent the number of samples in this area. In **(B)**, “Lineage A” is short for “haplotypes in Lineage A but not in Sublineage a1 in **(A)**,” while “Lineage B” was short for “haplotypes in Lineage B but not in Sublineage B1 and B2 in **(A)**.”

The COVID-19 outbreak in China coincides with the Spring Festival held in the country during 2020. Anhui is a populous province of China with frequent population movements and was significantly affected by this pandemic. As the capital of Anhui province, Hefei could not avert the spread of the coronavirus. Hefei were located in the central of Anhui Province (Figure 1), with a population of 9.3 million. From January 10 to March 10, 2020, the Hefei COVID-19 epidemic depicted the obvious characteristics of the global pandemic, and it was divided into three stages, namely, the early epidemic (January 10 to 23), the rapid rise period (From January 24 to 30), and the slow decline period (from January 31 to March 10). The first COVID-19 case reported in Hefei was from Wuhan and was confirmed on January 22. As of March 10, 2020, Hefei City has reported a total of 174 confirmed COVID-19 cases, thereby being the area with the highest number of cases in Anhui Province at that time. Among the 174 confirmed cases, 42 cases (24.14%) were in the early stage of the epidemic, 87 cases (50.00%) were in the rapid rise period, and 45 cases (25.86%) were in the slow decline period (35). During this time, a clinical isolate of SARS-CoV-2 virus from Hefei was cultured and examined by whole-genome sequencing. It was identified as the S-type strain. There were almost no observable variations in its sequence compared to the reference sequence available in the database at the time (36). This finding confirmed the relatively low variability of the SARS-CoV-2 genome. The whole-genome sequencing also revealed a group of mutations mainly located in the non-structural protein coding region (sites 2189, 3086, 5094, 8782, 11082, 16049, 17122, and 28144). It has been reported that mutations at 8782 and 28144 can divided SARS-CoV-2 into two types: L and S. The significance of other seven mutations in terms of virulence or disease severity is not clear (36).

In general, very little knowledge is available about the regional evolution of SARS-CoV-2 in Hefei. It can only be fulfilled by genome monitoring and analyzing whole-genome sequences of samples collected from Hefei during the epidemic.

The present study for the first time conducted whole-genome sequencing and molecular epidemiological analysis on 97 clinical samples from 29 COVID-19 inpatients of the First Affiliated Hospital of Anhui Medical University during the epidemic in Hefei. The genomic variations in SARS-CoV-2 strains of Hefei were revealed. The results helped assess the region-specific variation and frequency of single nucleotide polymorphism (SNP). Finally, our findings also determined the clinical characteristics of different genetic changes, with special attention to related immune responses to understand their potential impact on clinical outcomes.

## Materials and Methods

### Patients

The study protocols were approved by the Ethics Committee of the First Affiliated Hospital of Anhui Medical University (Quick -PJ 2021-12-17). A total of 97 oropharyngeal swab samples were collected from 29 patients who presented with COVID-19 at the First Affiliated Hospital of Anhui Medical University between January 28 and March 8, 2020. The First Affiliated Hospital of Anhui Medical University is comprehensive (first-rate of Level three) hospital and provides complete medical care and health services for patients across Hefei as well as surrounding areas in the Anhui province. It is one of the eight designated hospitals for treating COVID-19 patients in Hefei. At present, the hospital is equipped with 4,990 beds and the annual outpatient volume is about 5 million. Confirmation of COVID-19 and clinical classifications were based on the protocols outlined in the New Coronavirus Pneumonia Prevention and Control Program (4^th^ Edition), which was published by the National Health Commission of China (37). This program specifies that to be considered as a confirmed COVID-19 case, patients must have detectable SARS-CoV-2 RNA in at least one respiratory sample since illness onset and exhibit acute respiratory infection syndrome and/or abnormalities on computed tomography (CT) scan of the chest. All clinical data on epidemiology (including exposure history), symptoms, underlying comorbidities, and laboratory results were retrospectively extracted from electronic medical records. An illness was considered to be serious when a critical illness notice was present in the medical record.

### Detection of SARS-CoV-2 Using RT-PCR

Oropharyngeal specimens were obtained with flocked swabs and placed in universal transport medium (Beijing Youkang Technology, Beijing, China) at 4°C until processed. All stored samples were processed within 6 h. Nucleic acid was extracted using a viral RNA extraction kit (Da An Gene Co., Ltd. affiliated with Sun Yat-sen University, Guangzhou, China). The presence of SARS-CoV-2 (*N* and *ORF1ab* genes) was detected using a SARS-CoV-2 RT-PCR kit (Da An Gene Co., Ltd.) on an Applied Biosystems 7500 system (Thermo Fisher Scientific, Waltham, MA, USA), according to the manufacturer's instructions.

### SARS-CoV-2 Whole-Genome Sequencing

Genome sequences were determined for the 97 SARS-CoV-2 RNA samples isolated as described above. The whole-genome sequence of SARS-CoV-2 was amplified using the Ion AmpliSeq™ DNA custom Panel WG00428_Coronavirus (Thermo Fisher Scientific), containing two pools with 121 primer pairs each. Sequencing libraries were prepared with the AmpliSeq Library Kit 2.0 (Thermo Fisher Scientific) according to the manufacturer's instructions, with barcoding of each sample. PCR amplification was performed as follows: 99°C for 2 min, 26 times (99°C for 15 s and 60°C for 4 min), followed by a hold at 10°C. The PCR amplicons were treated with 2 μL FuPa reagent to partially digest the primer sequences and were phosphorylated at 50°C for 10 min, followed by 55°C for 10 min and 60°C for 20 min. The amplicons were then ligated to adapters with diluted barcodes using the Ion Xpress Barcode Adapters kit (Thermo Fisher Scientific) for 30 min at 22°C and then 72°C for 10 min, followed by purification of the adapter-ligated amplicons (library) using the Agencourt AMPure XP reagent (Beckman Coulter, Brea, CA, USA). Library concentration was evaluated using Real-Time PCR Systems. Each diluted library (100 pM) was amplified through emulsion PCR using the OneTouch™ Instrument (Thermo Fisher Scientific) and enriched with the OneTouch™ ES Instrument (Thermo Fisher Scientific) using the Ion PI Hi-Q OT2 200 kit following the manufacturer's instructions. Finally, sequencing was performed on an Ion Proton instrument (Thermo Fisher Scientific) using an Ion PI Hi-Q Sequencing 200 Kit (Thermo Fisher Scientific). For genome assembly that had reference sequences available, sequencing reads were mapped to the reference using Burrows–Wheeler aligner (Bwa, version 0.7.12-r1039). Reads with excessive variations, which suggest artifacts, were removed from the dataset. Reads with mapped lengths shorter than 30 bp were also removed, and the soft-clipped bases were trimmed from both ends. For ampliseq data, depth was sufficient; redundant/duplicate reads were removed accordingly. Finally, genome assembly was performed with trinity (v1.2.9) with default parameters MEGAHIT (v1.2.9) or with parameter –k-min 15 (38–40).

### Bioinformatic Analysis of Genome Sequencing

#### Analysis of SARS-CoV-2 Genomic Variation

Filtered reads were mapped to the Wuhan reference genome (GenBank ID: NC_045512.2) using the BWA software package, version 0.7.12-r1039, as described elsewhere (2, 23, 40). The total number of reads that were mapped to NC_045512.2 in each sample ranged from 5,818 and 26,187,027. The average coverage depth was between 5.13 and 160725.14. Using SAM tools, only variants with depths larger than 300 and quality scores larger than 30 were retained (24). Consensus sequences were constructed using both the reference genome and those called variants. Sequence alignment of consensus sequences obtained in this study and that of the reference genome was performed using MAFFT (v7.427) (41). The alignment was then imported into DnaSP 6 for sequence analyses (42).

To analyze regional distribution and phylogenesis, a total of 483 SARS-CoV-2 genome sequences from isolates identified in Eastern Asia and Southeast Asia were downloaded from NCBI (Supplementary Data 1). After removing sequences that were missing more than 2,000 consecutive bp, only 352 sequences were retained. Genome sequences obtained in this study and downloaded from NCBI were aligned with MAFFT and analyzed with DnaSP 6, as is standard practice for this method of analysis (41, 43).

### Phylogenetic Analysis of SARS-CoV-2 Sequences

A total of 231 genome sequences of betacoronaviruses isolated from mammals were downloaded from NCBI (Supplementary Data 1). The sequences were aligned with SARS-CoV-2 sequences obtained in this study using MAFFT. Phylogenetic trees were reconstructed using MEGA by Maximum Likelihood method. A bat coronavirus sequence (MN996532) was the most closely related sequence to SARS-CoV-2 and was thus used as an outgroup in subsequent analyses (44, 45). Since network reconstruction is extremely sensitive to missing data, only 48 samples (those missing <20% of the full-length genome sequence data) were included in the network analysis (Supplementary Data 1). To filter out rare mutations or alignment errors, network reconstructions used only parsimony-informative sites with binary polymorphisms. Two networks of haplotypes were generated in the NETWORK program using two data sets (46, 47). The first network used all the downloaded sequences from GenBank, as well as the sequences obtained in this study (Supplementary Data 1). Based on these results, sequences that did not distribute in the same branches as the study samples were removed, and the remaining sequences were used to create the second data set (Supplementary Data 1). In the NETWORK program, the median-joining network algorithm with the MP calculation option was used to reconstruct the most parsimonious network. Transversions were given a weight of three, while transitions were given a weight of one. In the network visualizations, nodes were proportional to frequencies of haplotypes and different colors indicated different regions. The network for the first data set was simplified using the star contraction option, as it was initially too complex to read. The data used in the first network was also used to reconstruct the phylogenetic tree of all SARS-CoV-2 sequences using MEGA by Maximum Likelihood method.

### Measurement of Inflammatory Factors

Levels of IL-6, IL-8, IL-10, IL-2R, and tumor necrosis factor-α (TNF-α) were assessed using a chemiluminescent immunoassay (Siemens, Munich, Germany); serum ferritin (SF) level was assessed using an electrochemiluminescence immunoassay (Roche, Mannheim, Germany) according to the manufacturer's instructions. The IMMULITE Cytokine Control Module, IMMULITE IL-10 Control Module (Siemens, Germany), and Lyphochek Immunoassay Plus Control (Bio-Rad, CA, USA) were used as internal controls, according to the manufacturer's instructions. The IMMULITE Cytokine Control Module is an assayed, bi-level control (containing different concentrations of selected lyophilized cytokines in a human serum matrix) intended for use with the IMMULITE1000 and IMMULITE 2000 IL-6, IMMULITE1000 IL-8, IMMULITE1000 IL-2R, and IMMULITE1000 TNFa assay. The IMMULITE IL-10 Control Module is a bi-level, synthetic matrix control intended for use with the IMMULITE1000 IL-10 assay. Lyphochek Immunoassay Plus Control (Bio-Rad) is a three-level, human serum matrix control intended for use with the Robas 6000 SF assay kit. The patients underwent multiple inflammatory factors tests during the hospitalization period. Based on the patient's conditions combined with the results of CT scan and nucleic acid test, the inflammatory factor data from samples obtained at the severest disease condition were selected for further analysis.

### Statistical Analysis

For each variable site, we divided patients into two groups: “reference” and “variation.” The “reference” group included patients carrying SARS-CoV-2 which was the same as reference genome at the site, whereas the “variation” group included patients carrying SARS-CoV-2 which mutated at the site. For 12 sites, both groups contained at least three patients, so totally 12 sites were used for subsequent analysis. The patients were also separated into “severe case” and “mild case” based on whether the medical record was present. Fisher's exact test was performed using SPSS software version 20.0 (IBM, Armonk, NY, USA) to test whether severe case percentage differed significantly (*p* < 0.05) between two mutation status. For each of the 12 sites, the expression levels of the six inflammatory factors between groups were compared using parametric *t-*tests using the SPSS software version 20.0 (IBM, Armonk, NY, USA).

## Results

### Identification of Genetic Variations in the Sampled SARS-CoV-2 Genomes

Our analysis of 97 SARS-CoV-2 RNA samples, derived from 29 COVID-19 patients at various time points, identified 263 SNPs, while no indels were found (summarized in Table 1, Supplementary Table 1). Thirty-five of the identified SNPs were parsimony-informative sites and 228 were singleton SNPs. This ratio of parsimony-informative sites to singleton SNPs is comparable to that found in human genomes (48). Notably, there were 188 novel sites found in the study samples that were absent from all SARS-CoV-2 genomes accessed from the NCBI database for this study. All but six of the discovered SNPs were assigned to protein-coding regions. Specifically, non-synonymous, synonymous, and nonsense mutations encompassed 167, 80, and 10 sites, respectively. This suggests a high tolerance for sequence variation at the function - primarily protein-level. Most strikingly, sites 10380, 18060, and 28144 exhibited the highest mutation frequencies of all sites and appeared to be hotspots for mutation. SNPs at sites 10380, 18060, and 28144 were found in 24, 29, and 43 isolates, respectively. Previous studies have also identified SNPs at sites 18060 and 28144 in SARS-CoV-2 isolates from other geographical locations (33, 49, 50). Moreover, a non-synonymous mutation at site 28144 can give rise to a Leu to Ser substitution in the ORF8 protein, whereas an SNP at site 18060 in *ORF1b* is functionally silent. However, the SNP at site 10380 (harboring a G → T mutation, Gly → Val) has not yet been reported in any other isolates and is likely to account for a novel variation to Hefei. Thus, it may serve as a useful marker in further large-scale molecular epidemiological studies.

**Table 1:** Counts of SNPs in the SARS-CoV-2 genomes analyzed in Hefei.

**Name**	**Counts of mutation**	**Counts of mutation**			**Counts of mutation**		**Counts of mutation**	
		**Synonymous**	**non-synonymous**	**Nonsense**	**Novel**	**Shared**	**Singleton variable**	**Parsimony informative**
Noncoding	6	\	\	\	2	4	6	0
ORF1a	112	35	71	6	85	27	99	13
ORF1b	71	20	48	3	55	16	62	9
S	21	6	15	0	11	10	19	2
ORF3a	14	3	11	0	9	5	12	2
E	2	1	1	0	2	0	2	0
M	6	4	2	0	5	1	4	2
ORF6	2	1	1	0	2	0	1	1
ORF7a	5	0	4	1	4	1	4	1
ORF7b	0	0	0	0	0	0	0	0
ORF8	5	3	2	0	2	3	2	3
N	18	6	12	0	10	8	16	2
ORF10	1	1	0	0	1	0	1	0

*The term “novel” means the mutation has not been identified in other SARS-CoV-2 sequences submitted to NCBI. The term “shared” means the mutation has also been identified in other SARS-CoV-2 sequences submitted to NCBI.

### Haplotype Network and Phylogenetic Analysis of SARS-CoV-2 Genome

Haplotype network and phylogenetic analysis were carried out to infer the evolutionary relationship between regional samples. Only sequencing data with > 80% total sequence coverage was included in the haplotype network reconstruction, so only 37 haplotypes were identified from the 48 samples used to reconstruct the network (Table 2). All the haplotypes were novel and had not been previously described; five of them were associated with multiple samples.

**Table 2:** SARS-CoV-2 haplotypes identified in Hefei.

**Patient**	**Illness severity**	**Date of enrollment**	**No. of sample**	**Allele of 28144**	**Allele of 8782**	**Counts of haplotypes in Network**
HF1	severe	Jan 26, 2020	9.20	T	C	2
HF2	mild	Jan 28, 2020	38	T	C	1
HF3	severe	Jan 28, 2020	37	T	C	1
HF4	severe	Jan 28, 2020	34.187	T	C	1
HF5	severe	Jan 28, 2020	29.179	T	C	2
HF6	severe	Jan 28, 2020	388	T	C	1
HF7	mild	Jan 30, 2020	60	T	C	1
HF8	severe	Jan 31, 2020	679.835	T	C	2
HF9	mild	Feb 6, 2020	300	T	C	1
HF10	mild	Feb 6, 2020	299	T	C	1
HF11	mild	Feb 7, 2020	240	T	C	1
HF12	severe	Feb 7, 2020	362.624	T	C	2
HF13	severe	Feb 7, 2020	241.396. 909	T	C	2
HF14	mild	Feb 7 2020	364	T	C	1
HF15	mild	Feb 7, 2020	363	T	C	1
HF16	severe	Jan 31, 2020	667	C	C	1
HF17	severe	Feb 2, 2020	153	C	T	1
HF18	severe	Feb 3, 2020	167	C	T	1
HF19	mild	Feb 7, 2020	247.626. 742	C	C	2
HF20	mild	Feb 7, 2020	619	C	T	1
HF21	mild	Feb 7, 2020	615.665. 806. 947	C	T	4
HF22	mild	Feb 12, 2020	425.543. 707. 839. 912. 932	C	T	4
HF23	mild	Feb 16, 2020	790	C	C	1
HF24	severe	Feb 16, 2020	764.795. 844	C	T	2
HF25	mild	Feb 18, 2020	661	C	T	1
HF26	mild	Feb 21, 2020	728.787. 863. 966	C	T	4

The whole haplotype network (Figure 1A) could be separated into two big lineages, which were labeled Lineage A and Lineage B. The core of Lineage B and that of Lineage A were distinguished by two mutations: the synonymous mutation T8782C and the non-synonymous mutation C28144T changing a leucine to a serine. These two lineages were well-known as L-type and S-type, respectively, as also reported in other network studies (43, 51). Both core haplotypes were super-spreaders and were distributed in almost all areas included in this study.

Lineage A (S-type) showed a closer relationship with the root (an animal virus sequence, MN996532) that represents the ancestral origin. Sequence MT079847 was used as the representative core haplotype for Lineage A. MT079847 differed from the reference sequence by only one synonymous mutation, C8782T, and one non-synonymous mutation, T28144C (Leu → Ser). Although Lineage A had transmitted to all the areas we included, it was mainly distributed in China. Within Lineage A, only one sublineage with more than five haplotypes was discovered, which was labeled a1 (Figure 1). All these haplotypes had a common synonymous mutation C18060T and were geographically exclusive to Hefei and Yangtze River Delta, thereby forming an endemic cohort.

Lineage B was derived from Lineage A, which consisted of more haplotypes and longer branches. The core haplotype of Lineage B was identical to the reference sequence NC_045512. Within Lineage B, two sublineage s, each with more than five haplotypes, were discovered. Sublineage B1, which had common mutations 26144 or 11083, was mainly distributed in Hong Kong and Southeast Asia. Sublineage B2, which had a common mutations 241, was mainly distributed in Hong Kong and Japan. Based on this analysis and data records, Sublineage B2 emerged more recently than Lineage A or the other Lineage B sublineages. Moreover, the overlap in the geographical distribution of Lineage A and Lineage B indicates frequent traveling as an important accelerating factor for the spread of infection.

Although Lineage A was more ancestral than Lineage B, their transmission to human and global circulation occurred synchronously (Figure 1B) and the genome of Lineage B was sequenced first (2). In Chinese mainland, both lineage s accounted for almost one half of the samples, however, Lineage B was represented at much higher frequency than Lineage A in Hong Kong and Japan. Especially, a newly emerging Sublineage B2, which was mainly isolated in most samples in Hong Kong and Japan, was rare in Chinese mainland. By contrast, Lineage a1 was local concentrated distribution in Hefei and the Yangtze River Delta, indicating that this cohort is an endemic S-type variation of the virus.

**Figure 2:**
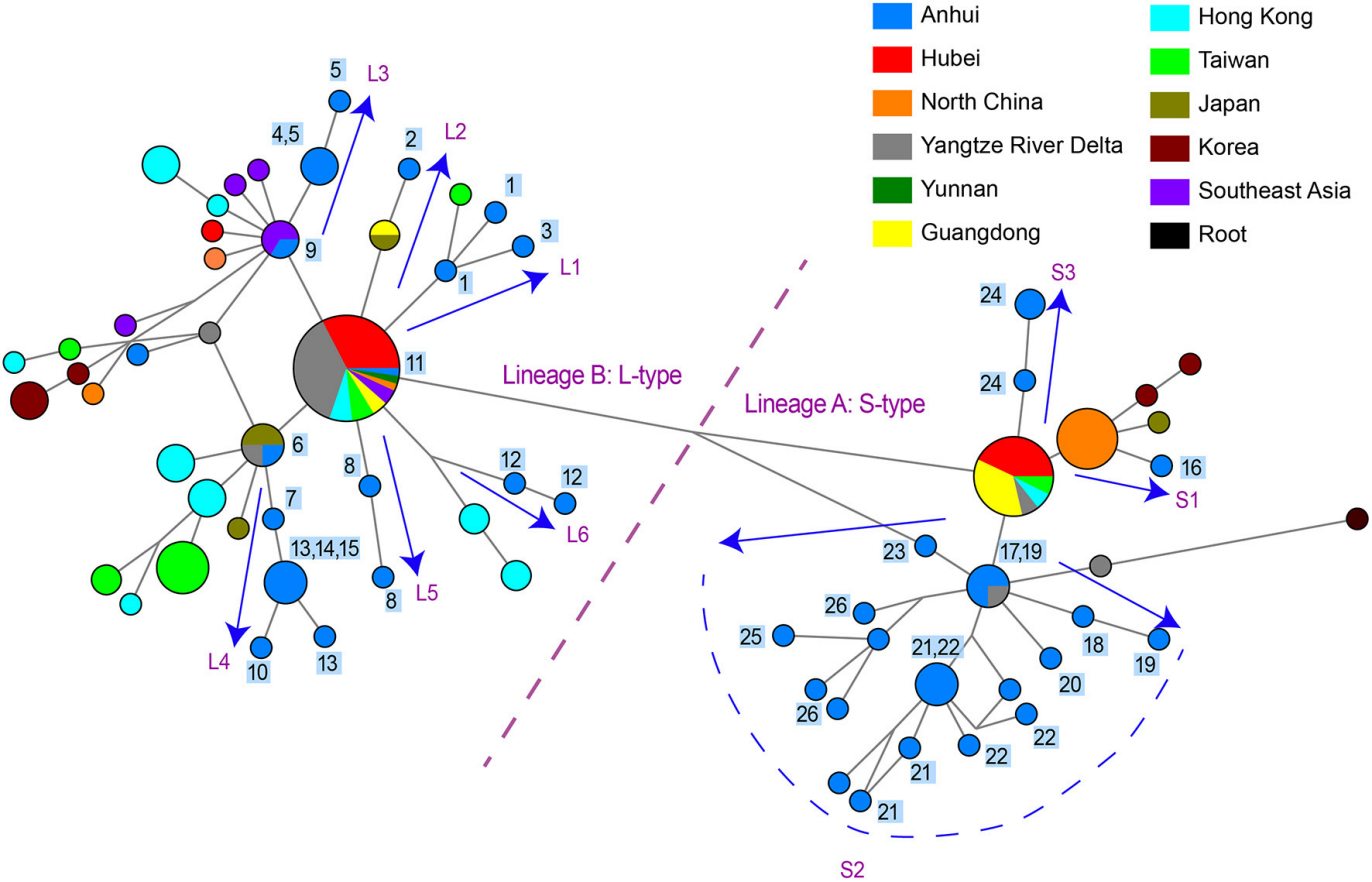
Phylogenetic network of 48 SARS-CoV-2 genomes in study samples and other sequences distributed in the same branches with them. Circle areas were proportional to the number of sequences and the edge linked two circle represent mutations between two sequences. Sequences collected from 26 patients in our hospital are labeled 1~26 next to the circles. The arrows showed the SARS-CoV-2 transmission paths to Hefei, labeled with L1~L6 for L-type and labeled with S1~S3 for S-type.

The results of phylogenetic tree (Supplementary Figure 1) showed the same results as network. All the sequence could be split into two Lineage A and B. Within Lineage A, a distinguished Sublineage labeled “a1” was limited in Hefei and Yangtze River Delta. Within Lineage B, two distinguished Sublineage labeled “B1” and “B2” were found. While Sublineage B1 was mainly distributed in Hong Kong and Southeast Asia. With a particular long branch, Sublineage B2 was transmitted in Japan quickly and extensively. The root (MN996532) had a very long branch, but it is nested in Sublineage a1. As for the samples collected in Hefei, they are distributed in nine different evolutionary branches in both phylogenetic network (Figure 2) and phylogenetic tree, indicting the introduction of SAR-CoV-2 to Hefei happened at least nine times.

### Reconstruction of Potential Infection Pathways in Hefei City

One practical application of phylogenetic network is to reconstruct potential pathways of human-to-human transmission of SARS-CoV-2. We investigated the transmission paths specifically associated with the infection in the Hefei region by focusing on locally obtained samples and comparing with other sequences distributed in same branches with them (Figure 2). The phylogenetic network analysis indicated that the initial infections likely occurred independently on at least nine separate occasions. Different viral types appeared to be introduced sequentially to the region, beginning with the L-type and followed by the S-type. A more detailed picture of potential transmission paths began to emerge when epidemiological data was incorporated into the analysis.

A minimum of six independent initial infections of the L-type virus likely occurred within a short period in January 2020 (labeled L1–L6 in Figure 2).

L1. Patient HF1 marked the first confirmed COVID-9 case admitted to our hospital. The patient traveled back from Wuhan, China on January 19, 2020, developed a fever on January 23, and was hospitalized on January 26. Patient HF3 had also returned from Wuhan and was hospitalized at approximately the same time as HF1. There was only one mutation that distinguished the HF1 isolate from the HF3, suggesting that HF1 and HF3 should be counted as one introduction event, which represents the earliest infections of the L-type SARS-CoV-2 in Hefei.

L2. Patient HF2 accounted for the second introduction event. The patient traveled back from Wuhan and was hospitalized on January 28, 2020.

L3. The third introduction occurred with patients HF4 and HF5. Both patients reported attending a social event with several individuals from Wuhan, soon developed COVID-19 symptoms, and were hospitalized on January 28. After 9 days, HF9 was admitted to our hospital. Although no direct contact was found between patient HF9 and patients HF4 and HF5, they shared similar haplotypes.

L4. The fourth introduction involved six cases (HF6, HF7, HF10, HF13, HF14, and HF15). Patient HF6 returned to Hefei from Wuhan on January 21 and was admitted to the hospital on January 28 with serious conditions. Soon after, patients HF7, HF10, HF13, HF14, and HF15 were hospitalized. Notably, patients HF10, HF13, and HF14 were from the same family and had no historical direct contact with anybody in Wuhan. This suggests the possibility of human-human transmission, a theory supported by evidence that one of the two viral haplotypes identified in the HF13 isolate was shared by HF14 and that the sequences of the HF10 and HF14 isolates differed by a single nucleotide.

L5 and L6. HF8 and HF12 account for two separate introduction events.

During the same period in early 2020, the S-type SARS-CoV-2 was introduced into Hefei on at least three independent occasions (labeled S1–3 in Figure 2).

S1. HF16 represented the first introduction, which occurred on January 21 when the patient-already exhibiting serious symptoms-traveled back to Hefei from Wuhan. The viral haplotype of this isolate has no direct relationship to any other case of S-type infections under study and was thus defined as a separate introduction.

S2. The second introduction involved nine individual cases (HF17, HF18, HF19, HF20, HF21, HF22, HF23, HF25, and HF26). With no clear historical direct contact with infection cases in Wuhan, patients HF17 and HF18 were admitted to the hospital on February 2 and 3, respectively, and represent the earliest patients in S2 (Figure 2) within our healthcare system. The viral haplotypes of the HF17 and HF18 isolates were differed by only one mutation and were closely associated with all other isolates of this cohort. Notably, HF21 and HF22 cases occurred in the same household, which also indicate a pattern of human-to-human viral transmission within this cohort. This second introduction of an S-type SARS-CoV-2 was responsible for nine individual cases involving viral haplotypes that shared a synonymous mutation, C18060T. These haplotypes constitute the majority of Sublineage *a*1, as highlighted in the haplotype network analysis (Figure 1A), which appears to be concentrated in Hefei and the Yangtze River Delta region, indicating that this cohort is an endemic S-type variant of the virus.

S3. HF24 accounted for the third S-type introduction event and was distinguished from other S-type isolates.

### Mutational Sites Were Not Associated With Disease Severity

As previously mentioned, an illness was only considered to be serious if a critical illness notice was present in the medical record. The level of inflammatory factors and the grouping information for each patient are summarized in Supplementary Table 2. In total, 12 sites (8782, 10380, 11083, 13394, 14418, 16954, 17614, 18060, 26144, 26885, 28144, and 28253) were selected for a Fisher's exact test for comparing the rate of severe cases among different mutation status (Table 3). In this regard, no significant differences were observed among these sites, suggesting that the differences in mutation status might contribute to other viral functions and properties, such as transmissivity, or the sample size was insufficient to infer significant results.

**Table 3:** Difference in rates of severe cases between different mutation status.

**Site**	**Severe cases in VG**	**Mild cases in VG**	**Rate of severe cases in VG**	**Severe cases in RG**	**Mild cases in RG**	**Rate of severe cases in RG**	**Fisher's exact test**
8782	3	6	0.33	9	11	0.45	0.694
10380	4	10	0.29	8	7	0.53	0.264
11083	2	5	0.29	10	12	0.45	0.665
13394	2	3	0.4	10	14	0.42	1
14418	1	4	0.2	11	13	0.46	0.37
16954	0	3	0	12	14	0.46	0.246
17614	1	4	0.2	11	13	0.46	0.37
18060	2	9	0.18	10	8	0.56	0.064
26144	2	1	0.67	10	16	0.38	0.553
26885	1	3	0.25	11	14	0.44	0.622
28144	4	11	0.27	8	6	0.57	0.139
28253	3	1	0.75	9	16	0.36	0.279

*Based on the mutation status on each site, we divided the patients into two groups. VG represent a group of patients carrying COVID-19 which mutated on this site, while RG represent a group of patients carrying COVID-19 which was the same as reference at this site.

### Site 18060 and 28253 Mutations Correspond to Milder Immune Responses in Patients

**Figure 3:**
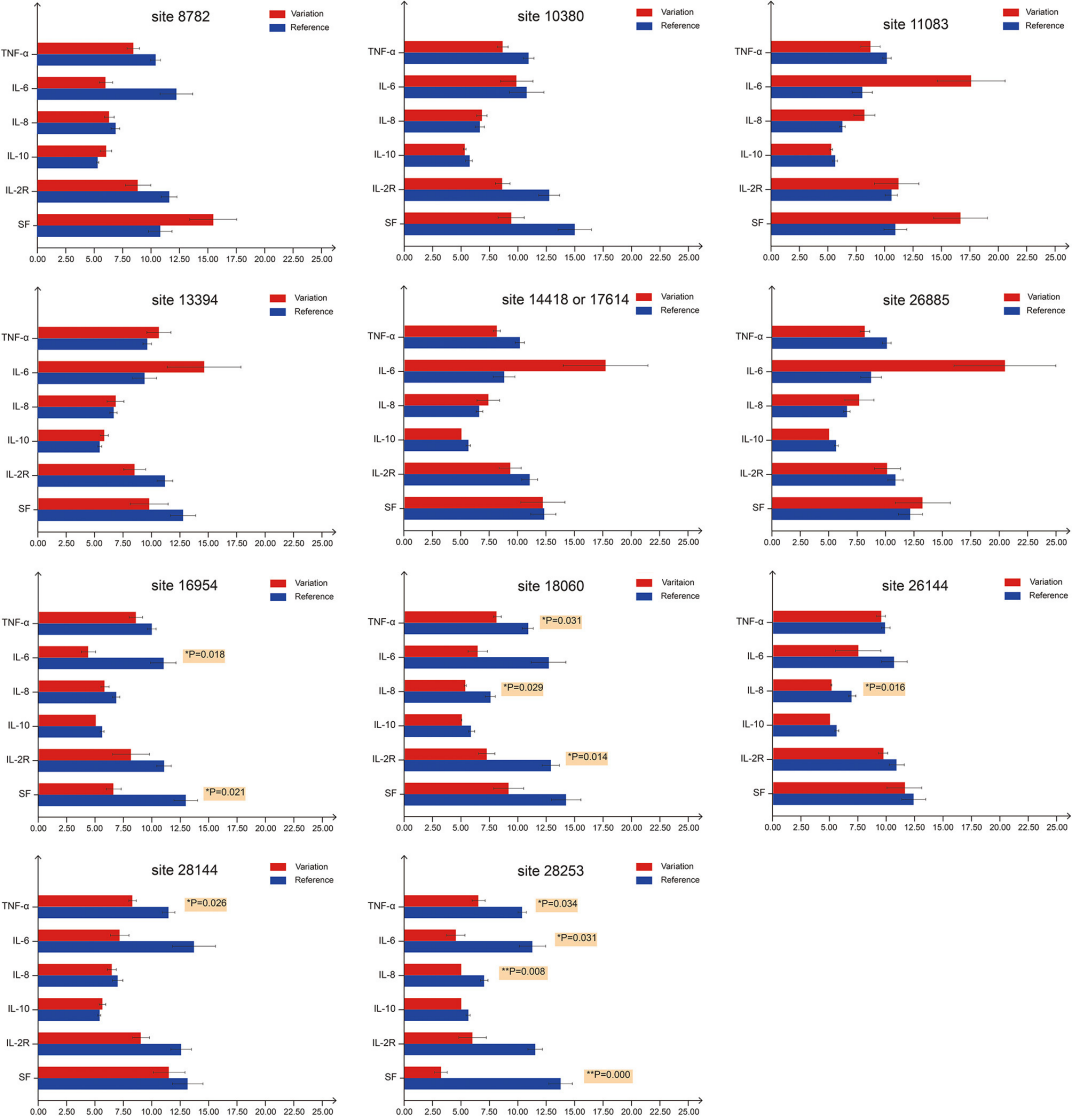
Serum levels of inflammatory factors in COVID-19 patients with different mutation status. For the first bar chart, the patients were divided into two groups based on the mutation status on site 8782. Red bars represent patients carrying SARS-CoV-2 with mutations at site 8782, whereas blue bars represent patients carrying SARS-CoV-2 that is the same as reference genome at site 8782. TNF-α, IL-6, IL-8 and IL-10 levels are expressed in 1 ng/mL units, while IL-2R and SF in 50 ng/mL units. The 11 bar charts represent sites 8782, 10380, 11083, 13394, 14418/17614, 26885, 16954, 18060, 26144, 28144, and 28253. *p-*values on the right of the bar denote significant differences (*p* < 0.05) between two groups. For sites 18060 and 28253, at least three inflammatory factors show significant differences between groups.

Recent reports indicate that the initiation and progress of the sickness caused by COVID-19 are driven by cytokine (e.g., IL-6 and IL-8) responses. Thus, therapy that targets cytokines may improve the health of COVID-19 patients (9). Similarly, numerous studies have shown that high SF level plays a key role in inflammation and is significantly correlated to the severity of the disease (52). Therefore, we investigated whether mutation status at genomic sites (Table 3) could affect serum concentrations of inflammatory factors (Figure 3). We monitored levels of several inflammatory factors, including IL-6, IL-8, IL-10, IL-2R, TNF-α, and SF, in the patient blood samples and found that inflammatory responses were significantly reduced (*p* < 0.05) in patients infected with the isolates bearing mutations at sites 18060(harboring a C → T synonymous mutation, Leu) and 28253 (harboring a C → T synonymous mutation, Phe). Basing on these findings, we speculated that these mutations, especially those at 18060 (i.e., the local endemic variation), may facilitate viral transmission and contribute to global public health initiatives by exposing populations to asymptomatic or mildly symptomatic versions of this deadly virus.

## Discussion

RNA viruses are often characterized by high mutation rates that often result in changes to characteristics such as virulence, host entry efficiency, receptor binding affinity, and transmission routes (53, 54). Discovery and identification of mutations in the SARS-CoV-2 genome is critical for not only understanding the infection mechanism but also for tracking the evolution and transmission of the virus (32, 55, 56).

Despite recent discovery of numerous variants, overall genomic variations in the SARS-CoV-2 seems generally low (57, 58). This characteristic was also found in our comparison of 97 SARS-CoV-2 genomic sequences, which revealed that an average of over 99.9% inter-sample sequence identity. However, specific hot spots within the genome display higher variability and are closely associated with various key aspects of the infection (50). For example, the variation in *ORF1a, ORF1b, S, N*, and *ORF8* genes appear to affect host infection and virus transmission by facilitating the adaptation and propagation of the virus in host cells. Specifically, the region located between *ORF1a* and *ORF1b* encodes the RNA-dependent RNA polymerase (RdRp) that is critical for viral gene expression. This region is used widely to clinically diagnose SARS-CoV-2 infections using PCR testing (2, 55, 59).

When the COVID-19 outbreak began in Hefei in January 2020, a set of clinical isolates were collected over time and whole-genome sequenced to identify a collection of variations at the mutation hot spots (Table 1). The G10380T mutation was exclusively associated with the isolates from Hefei, suggesting a endemic genetic variation in Hefei. This mutation was identified in 24 out of 97 samples studied, but only two samples could recover >80% of full-length genome sequence. This low sequence coverage is likely due to greater RNA instability as a result of the glycine/valine conversion introduced by the G10380T mutation. Perhaps G10380T was a global variation, which was unable to be detected due to chemical instability.

The SARS-CoV-2 strains have been categorized as L- and S-types (50), labeled with Lineage B and A in Figure 1A, respectively. The S-type is evolutionarily more ancient and likely gave rise to the L-type. The L-type predominates in the overall population accounting for 70% of known infections and is characterized by higher mutation rates than the S-type. A positive feedback loop is hypothesized in which the L-type can rapidly spread within a population due to increased infection and proliferation efficiency, allowing it to accumulate higher levels of mutations that can potentially enhance various viral capabilities (50, 55). In the early stages of the outbreak in Wuhan (Figure 1), both L-type and S-type SARS-CoV-2 were transmitted concurrently, but L-type accounted for most of the disease severity. In Hefei, early isolates from the COVID-19 cases were primarily the L-type virus, and the patients developed serious illness upon hospital admission. However, the S-type virus was introduced to Hefei later, and the patients typically displayed milder symptoms. The scenario in Hefei suggested that L-type was associated with faster transmission and severer symptoms than S-type, possibly explaining as to why L-type is predominant globally.

The haplotype network of SARS-CoV-2 reconstructed in this study was similar to earlier studies (43, 50, 51). All these studies found the C8782T and T28144C separated the entire network, and S-type was closely connected to the outgroup. For S-type, Forster et al. discovered a very big subcluster with a mutation on 29095, which formed a loop with another mutation on 18060 (36, 51). As our study was restricted to samples within Asia, we only found these two sublineage s contained in samples from distantly related areas and did not find a loop between the two branches (Figure 1A). For L-type, Forster et al. discovered a very big subcluster with mutation on 26144 and formed a loop with another mutation on 11083, which was the same as our results (Sublineage B1) (51). As for Lineage B2 (Figure 1A), which was a very small branch in the study by Forster et al. (51) it could be explained that these haplotypes much recently emerged and expanded. Although network analysis is potentially affected by distinctive migratory histories, founder events, and sample size, repeatability was considerable for the huge number of SARS-CoV-2 sequences deposited in public databases. Moreover, the lineages of phylogenetic tree (Supplementary Figure 1) were also separated into Lineage A and B, with Sublineage a1 nested in Lineage A, and Sublineage B1 and B2 nested in Lineage B. Different analysis method could lead to convergence, indicating the data was robust.

Considering the chronological, geographical, and clinical aspects of all the cases under study, we propose the following possibility for understanding the development of the COVID-19 epidemic in Hefei: Direct contact with cases from Wuhan likely led to the majority of cases. In this narrow window of time (from Jan 26 to Feb 21), the L-type was introduced to Hefei first, generally exhibiting a higher level of virulence, and S-type emerged later. Meanwhile, comprehensive prevention and control measures were created and enforced in an attempt to restrict viral transmission and slow down the impacts of the epidemic. Patients carrying the L- type SARS-COV-2 were more likely to develop clinical symptoms and were thus more susceptible to medical intervention. Partially as a result, the S-type strain, particularly the Sublineage *a1* (C18060T) variant, became endemic to Hefei and the Yangtze River Delta region and tended to cause less severe clinical symptoms. This shift in the mode of transmission, along with the evolution of the virus, suggests that the SARS-COV-2 may eventually settle into a niche area as a mild and periodic viral pathogen similar to influenza virus. Even then, the virus would still carry the alarming potential to cause an epidemic or a pandemic.

Contrary to our hypothesis, viral mutations had no significant effect on the rate of severe cases, even though a significant number of mutations were non-synonymous. Potential biases may have arisen from the limitations in both sample size and the amount of time for clinical observation. However, the levels of inflammatory factors (IL-6, IL-8, TNF-α, IL-6, IL-2R, and SF) in serum samples were significantly decreased in patients infected with isolates bearing mutations at positions 18060 (*ORF1ab*) and 28253 (*ORF8*). The C18060T mutation was exactly the distinctive site using to distinguish the local Sublineage (a1) of S-type SARS-CoV-2 endemic to Hefei and the Yangtze River Delta region. Notably, these were synonymous mutations, signifying that they may lead to functional shifts due to codon bias in translation which may affect to the efficiency of protein translation. Codon preference may contribute to the efficiency of protein translation. Frequently used codons correspond to abundant tRNAs, and tRNA content directly affects the rate of amino acid translation. Different organisms use various codons at different frequencies and various biological codon usage preference data can be found in the Kazusa Codon Usage Database (kazusa.or.jp/codon/) (60, 61). In human codons, the frequency of CTC is 19.6%, while the frequency of CTT is 13.2%. The frequency of TTC is 20.3%, and the frequency of TTT is 17.6%. Thus, the mutations at sites 18060 (CTC → CTT) and 28253 (TTC → TTT) may affect the rate of protein synthesis. Through genomic surveillance, we identified a locally concentrated S-type SARS-CoV-2 Sublineage, *a1* (C18060T), that was endemic to Hefei and the Yangtze River Delta region, thus providing important insights into the local development of COVID-19. Our work highlights the importance of genomic surveillance for understanding and controlling pandemics, as well as the potential value of following the dynamic shifts of viral subtypes when studying the transmission, pathogenicity, and evolution of the viruses.

The limitation of this study is that sample size was not large enough. There were only 42 patients diagnosed as having COVID-19 in our hospital during local outbreak in 2020. According to the experimental purpose and methods, some patients with incomplete data were removed from study, bringing down the final number of evaluable cases to 29. The experimental data were collected from January 28 to March 8, 2020. The time coverage of all the COVID-19 patients included in this study was 41 days. During this period, several nucleic acid tests were performed based on the patient conditions. Consequently, the number and timing of nucleic acid test was different for each patient. Therefore, the time-dependent shift in mutation profile was not investigated. In the past year, the number of COVID-19 cases in Hefei has remained zero although the delta variant has been prevalent in many other areas. We are currently unable to expand the sample size to verify the existing conclusions. We plan to conduct further mechanistic studies based on the existing sample data.

Conclusively, the present study revealed the endemic variations of SARS-CoV-2 in Hefei, for the first time, which may be related to the milder, local COVID-19 epidemic. Expanding the ongoing sequencing efforts to monitor SARS-CoV-2 subtype will be critical in identifying future variants of concern and understanding the mechanisms of innate immune evasion by which SARS-CoV-2 adapts to a new host environment.

## Data Availability Statement

The datasets presented in this study can be found in NCBI GeneBank under accession number MZ824620 to MZ824664.

## Ethics Statement

This study protocols were approved by the Ethics Committee of the First Affiliated Hospital of Anhui Medical University (Quick -PJ 2021-12-17). Written informed consent for participation was not required for this study in accordance with the national legislation and the institutional requirements.

## Author Contributions

Y-hX and H-qZ contributed to the conception, design of experiments, gave final approval, and agreed to be accountable for all aspects of work ensuring integrity and accuracy. X-wC, JL, LZh, and NZ drafted and critically revised the article. X-wC, JL, W-jH, LZo, XX, J-pQ, M-jZ, X-wJ, and Z-kL completed all experiments. X-wC, LZo, Y-fZ, X-wJ, and Z-kL contributed to the analyses. All authors have read and agreed to the published version of the manuscript.

## Funding

This study has received funding from the Emergency Project of Anhui Medical University of Science and Technology (Grant No. YJGG202002), Anhui Medical University Scientific Research Fund (Grant No. 2020xkj165), and the National Natural Science Foundation of China (Grant No.31700286, 82072492, and 81501814).

## Conflict of Interest

X-wJ is employed by Daan Gene Co., Ltd. Z-kL is employed by Guangzhou Darui Biotechnology Co., Ltd. The remaining authors declare that the research was conducted in the absence of any commercial or financial relationships that could be construed as a potentialconflict of interest.

## Publisher's Note

All claims expressed in this article are solely those of the authors and do not necessarily represent those of their affiliated organizations, or those of the publisher, the editors and the reviewers. Any product that may be evaluated in this article, or claim that may be made by its manufacturer, is not guaranteed or endorsed by the publisher.
