# Supplementary material for: Identification of SARS-CoV-2 Variants and Their Clinical Significance in Hefei, China
Source: Front Med (Lausanne). 2022 Jan 10;8:784632. doi: 10.3389/fmed.2021.784632 (PMC8784789; doi:10.3389/fmed.2021.784632)
Supplement: Supplementary file 5 [file Presentation_1.pdf]

Supplementary Figure 1. Phylogeny of SARS-CoV-2 genomes generated by Maximum Likelihood method. Different color of each sequence ID represent different region of origin for each isolate. Two big lineages labeled A and B were discovered. In Lineage A, one Sublineage labeled a1 was distinguished from other members in the same lineage. Meanwhile, in Lineage B, Sublineage B1 and Sublineage B2 were distinguished, and Sublineage B2 had a extraordinary long branch transmitted mainly in Hong Kong and Japan. Sublineage a1 was formed of only sequences isolated in Hefei and Yangtze River Delta. The root (MN996532) had a very long branch, but it is nested in Sublineage a1. The isolates collected in Hefei were distributed in nine different branches, indicating independent introduction happened at least nine times.
